# Immunological mechanisms underlying progression of chronic wounds in recessive dystrophic epidermolysis bullosa

**DOI:** 10.1111/exd.14411

**Published:** 2021-06-27

**Authors:** Leonie Huitema, Taylor Phillips, Vitali Alexeev, Olga Igoucheva

**Affiliations:** ^1^ Department of Dermatology and Cutaneous Biology Sidney Kimmel Medical College Thomas Jefferson University Philadelphia PA USA

**Keywords:** cutaneous disease, epidermolysis bullosa, immunity, inflammation, microbiota, wound healing

## Abstract

Hereditary epidermolysis bullosa (EB) is a mechanobullous skin fragility disorder characterized by defective epithelial adhesion, leading to mechanical stress‐induced skin blistering. Based on the level of tissue separation within the dermal‐epidermal junction, EB is categorized into simplex (EBS), junctional (JEB), dystrophic (DEB) and Kindler syndrome. There is no cure for EB, and painful chronic cutaneous wounds are one of the major complications in recessive (RDEB) patients. Although RDEB is considered a cutaneous disease, recent data support the underlying systemic immunological defects. Furthermore, chronic wounds are often colonized with pathogenic microbiota, leading to excessive inflammation and altered wound healing. Consequently, patients with RDEB suffer from a painful sensation of chronic, cutaneous itching/burning and an endless battle with bacterial infections. To improve their quality of life and life expectancy, it is important to prevent cutaneous infections, dampen chronic inflammation and stimulate wound healing. A clear scientific understanding of the immunological events underlying the maintenance of chronic poorly healing wounds in RDEB patients is necessary to improve disease management and better understand other wound healing disorders. In this review, we summarize current knowledge of the role of professional phagocytes, such as neutrophils, macrophages and dendritic cells, the role of T‐cell‐mediated immunity in lymphoid organs, and the association of microbiota with poor wound healing in RDEB. We conclude that RDEB patients have an underlying immunity defect that seems to affect antibacterial immunity.

## 1 INTRODUCTION

Epidermolysis bullosa (EB) is a heterogeneous group of inherited skin disorders that affects approximately 19 per million live births, with 8 per million U.S. habitants having EB.^1^ Based on the skin phenotype of EB patients and the function of the EB‐associated gene variants, EB is considered a mechanobullous disorder. It is characterized by defective epithelial adhesion leading to skin fragility and the development of skin lesions following minor stress to the skin.^2^ Body sites naturally exposed to relatively minor mechanical stress or friction, such as knees, elbows, feet and hands, are prone to blistering. Significant progress has been made in understanding the molecular genetics of EB as well as the development of different therapies.^3^, ^4^, ^5^ Currently, 21 different disease‐causing gene variants associate with an EB phenotype.^2^, ^3^, ^6^ The type and combination of the mutations in identified genes result in a spectrum of phenotypic severity of EB, which varies from early lethal to mild variants with normal life expectancy. EB divides into 4 categories: EB simplex (EBS, common mutated genes are keratin [*KRT5* and *KRT14*]); junctional EB (JEB, the common mutated genes are *LAMA3*, *LAMB3*, *LAMC2* and *Col17*); dystrophic EB (DEB, the common mutated gene is *Col7A1*); and Kindler EB (KEB; the common mutated gene is *FERMT1*).^2^, ^3^, ^6^ Identified polymorphic genes found in EB patients all encode proteins essential for maintaining the integrity and structure of the skin and mucosa.^2^ On average, ~40% of EB patients are diagnosed with EBS, ~25% with JEB, ~35% with DEB and ~0.4% with Kindler syndrome.^1^

In patients with recessive DEB (RDEB), in particular, continuous blistering, inflammation, relapsing infections and disturbed regeneration lead to painful, chronically inflamed often non‐healing wounds. Furthermore, colonization and invasion of wounds with pathogenic bacteria result in excessive inflammation and affect wound healing progression.^1^, ^7^ This makes RDEB patients prone to life‐threatening infections and sepsis, a leading cause of infant mortality.^8^, ^9^ For RDEB survivors, a painful sensation of inflammatory cutaneous burning severely impedes quality of life. Additionally, repeated minor mechanical stress before the wound‐healing process is complete limits the potential for cutaneous regeneration. Consequently, over time, these chronic wounds are accompanied by fibrosis, scarring, mitten deformities and, frequently, a deadly aggressive form of metastatic squamous cell carcinoma (SCC; >90% by the age of 55 years).^10^, ^11^

Recently, a number of investigations have focussed on the immunological aspects of EB, and accumulating studies now support the hypothesis of immunological mechanisms underlying the mucocutaneous manifestation.^7^, ^10^, ^12^, ^13^, ^14^ Most common EB‐causing gene variants, such as KRT5, KRT14, LAMA3, LAMB3, LAMC2 and Col7A1, are also expressed in lymphoid organs,^12^, ^15^, ^16^ suggesting that dysfunction of these gene products could also affect the immune system and that EB patients may have a systemic immunity defect. In line with this, studies have also provided evidence for an intrinsic pro‐inflammatory state in EB patients. For example, high levels of cytokines, such as interleukin (IL)‐1β, IL‐2 and IL‐6, have been observed in the serum of EB patients.^13^

There is no cure for EB, and painful chronically inflamed wounds are one of the major complications.^17^ These wounds are predisposed to infection, which can ultimately lead to sepsis. Importantly, the high predisposition of cutaneous infections may be due to a systemic immunological defect that consequently affects antibacterial immunity. Therefore, additional knowledge is urgently needed about the role of EB‐related gene variants in central and peripheral lymphoid organs as well as the immunological state of EB‐affected patients. Subsequently, systemic therapeutic targeting of systemic inflammatory mediators in combination with regenerative medicine and/or conventional antibiotics could be an effective therapeutic strategy to alleviate disease manifestations. Taken together, the ability to regulate chronic wound inflammation and promote physiological healing of skin wounds would improve the quality of life and the life expectancy of patients. In this review, we will discuss potential immunological mechanisms underlying the maintenance and progression of wounds in RDEB patients.

## 2 THE ROLE OF PROFESSIONAL PHAGOCYTES IN EB WOUNDS

Physiological wound healing is divided into four phases: haemostasis, inflammation, proliferation and maturation. An acute inflammatory response is induced immediately when a wound is established during the haemostasis phase. This phase starts with coagulation of blood by platelets in the ruptured blood vessels and the release of signalling molecules, such as inflammatory cytokines, transforming growth factor‐beta (TGF‐β) and platelet‐derived growth factor (PDGF) that recruit innate immune cells, such as neutrophils, macrophages and dendritic cells (DC).^18^, ^19^

### 2.1 Neutrophils

Neutrophils are the first professional phagocytes to arrive at the site of a cutaneous wound, and they constitute about 50% of all cell types in wounds at day 1 after injury.^19^ Neutrophils exist in a resting state in the blood flow, but they become activated in response to cytokines and chemokines such as tumor necrosis factor‐alpha (TNFα), granulocyte‐macrophage colony‐stimulating factor (GM‐CSF), interleukin (IL)‐8, interferon‐γ (IFNγ) and bacterial products such as lipopolysaccharide (LPS).^20^ Their primary function is to prevent infection by attacking any pathogen invading the body via the open wound. Therefore, neutrophils produce antimicrobial peptides, reactive oxygen species, proteases and neutrophil extracellular traps.^21^ During or after the inflammatory stimulus, neutrophils undergo apoptosis. Apoptotic neutrophils are then engulfed by macrophages, which come into wounds in a second wave of cellular recruitment and down‐modulate the acute inflammatory response.^20^, ^21^

The enzymes and chemicals that the neutrophils produce to kill pathogens are non‐specific and often associated with collateral tissue damage, leading to delayed healing and excessive scar formation.^21^ Furthermore, extensive neutrophil infiltration and activation contribute to tissue injury and chronic inflammation.^20^ A dense epidermal neutrophilic infiltrate has been observed in biopsies of blistered skin from EBS patients.^22^ Furthermore, our group found that neutrophils are constitutively present and represent the majority of leucocytes (up to 90%) in chronic wounds of RDEB patients.^7^ These findings indicate that excessive neutrophilic infiltrate could be responsible for RDEB‐associated wound healing defects.

Blister fluids of DEB and JEB patients contain increased levels of IL‐8 and matrix metalloproteinase 9 (MMP9), which is a granular protease in neutrophils.^23^, ^24^ Recently, we have also found that MMP9 and cathepsin G activity are upregulated in established and chronic RDEB wounds.^7^ MMP9 and cathepsin G are the main granular proteases in neutrophils^20^ and neutrophil‐derived proteases associated with chronic non‐healing wounds.^21^ Mechanistically, these proteins degrade extracellular matrix components, and their excess may prevent proper wound closure in chronic wounds. Therefore, pharmacological targeting of matrix‐remodelling enzymes in chronic wounds may be a future strategy to enhance healing in DEB patients.

Potential drugs that dampen neutrophil recruitment towards cutaneous wounds of RDEB patients could suppress neutrophil‐dependent tissue damage. Chemokines and chemokine receptors regulate neutrophil recruitment to the wound site.^19^ Our comprehensive chemokine analysis of blister fluids from RDEB‐affected wounds revealed a high level of the ligands for CXCR 1, CXCR2, CCR2 and CCR4 chemokine receptors, as well as substantial infiltration of CXCR2^+^ CD11b CD16^+^ neutrophils.^14^ Therefore, CXCR2 antagonists could be used for targeted interruption of neutrophil infiltration in wounds of RDEB patients.^20^ Increased recruitment of neutrophils is also observed into chronically inflamed lungs of cystic fibrosis (CF) patients and chronic obstructive pulmonary disease (COPD).^25^ It has been shown that the CXCR2 antagonist, SB‐656933, inhibits the recruitment of neutrophils to the lungs in CF patients.^26^ In line with this, CXCR2 antagonists reduces airway neutrophilia in inflammatory airway models of mice, rats and monkeys.^27^ Clinical trials with CXCR2 inhibitors for airway inflammatory diseases are currently ongoing.^25^, ^28^ However, a major concern about inhibiting neutrophil recruitment is that this induces immunosuppression,^25^ which should be avoided for effective antimicrobial immunity in patients.

In addition to the understanding of the molecular mechanisms controlling increased neutrophil recruitment to chronic RDEB wounds,^7^, ^14^ additional knowledge is required about the phagocytic function and bacterial killing capacity of neutrophils present in wound beds. Previously, we have investigated the expression of complement component 5a receptor 1 (C5aR1) and C5L2 on CD66B^+^ neutrophils,^7^ as down‐modulation of these receptors correlates with a reduced phagocytic capacity.^29^ However, we did not observe significant changes in the cell surface expression of these receptors on CD66^+^ neutrophils in different RDEB‐derived wound types,^7^ and neutrophil activity in wounds remains to be further investigated. Taken together, current data suggest that in RDEB wounds, neutrophils should be regulated but not be completely “wiped out” as neutrophils are an important first‐line defense against pathogenic microbiota and are needed for proper matrix remodelling. Additionally, neutrophils communicate with other immune cells,^20^ and inhibition of neutrophil wound recruitment could skew immune response. Therefore, it is important to gain knowledge about how chronic wound‐derived neutrophils communicate with other immune cells and whether defects in neutrophil function are intrinsic or a consequence of another systemic immunological defect.

### 2.2 Macrophages

Skin‐resident macrophages are the most frequent immune cell type in the dermis. In response to local skin injury, resident macrophages and monocytes migrate to the affected site. Once arrived, monocytes differentiate into pro‐inflammatory macrophages in response to locally produced inflammatory cytokines.^30^ As the inflammatory response progresses, macrophages become the dominant professional phagocyte after 24–72 h of injury.^18^ Macrophages have multiple functions, including antimicrobial action, phagocytosis of aged neutrophils, debridement and wound regulation. Without macrophages, wound healing would not progress.^18^, ^31^, ^32^ Our prior study showed that, in RDEB patients, the percentage of macrophages in wound bed‐associated leucocytes drops significantly in chronic wounds compared with early and established wounds.^7^ An impaired macrophage response towards cutaneous bacterial infection has also been shown in DEB mice.^12^ The low percentage of macrophages may explain the prevalence of neutrophils in the wound bed and the lack of proper control of the inflammatory stage of wound healing in RDEB patients.^20^

The low percentage of macrophages in RDEB wounds^7^ suggests an intrinsic, innate immune defect due to the absence or dysfunction of type VII collagen (Col7). Nyström et al. (2018) support this hypothesis by showing that Col7 binds cochlin, the innate immune activator, in draining lymph nodes.^12^ The enzyme aggrecanase cleaves cochlin in response to bacterial infection, releasing the cochlin LCCL domain into the bloodstream,^33^ and the cochlin LCCL domain activates macrophages.^12^ Importantly, cochlin‐deficient mice fail to clear *Pseudomonas aeruginosa* and *Staphylococcus aureus* lung infections.^34^ Furthermore, cochlin LCCL is diminished in RDEB (col7‐deficient) mice and patients during bacterial infections. Systemic administration of cochlin LCCL domain to RDEB mice decreases cutaneous bacterial colonization.^12^ This implies that lack or dysfunction of Col7 directly affects innate immunity against pathogenic microbiota. Therefore, the Col7‐cochlin axis in lymphoid organs is a promising therapeutic target in RDEB, and mechanistic players within this axis should be further investigated.

### 2.3 Dendritic cells

**FIGURE 1:**
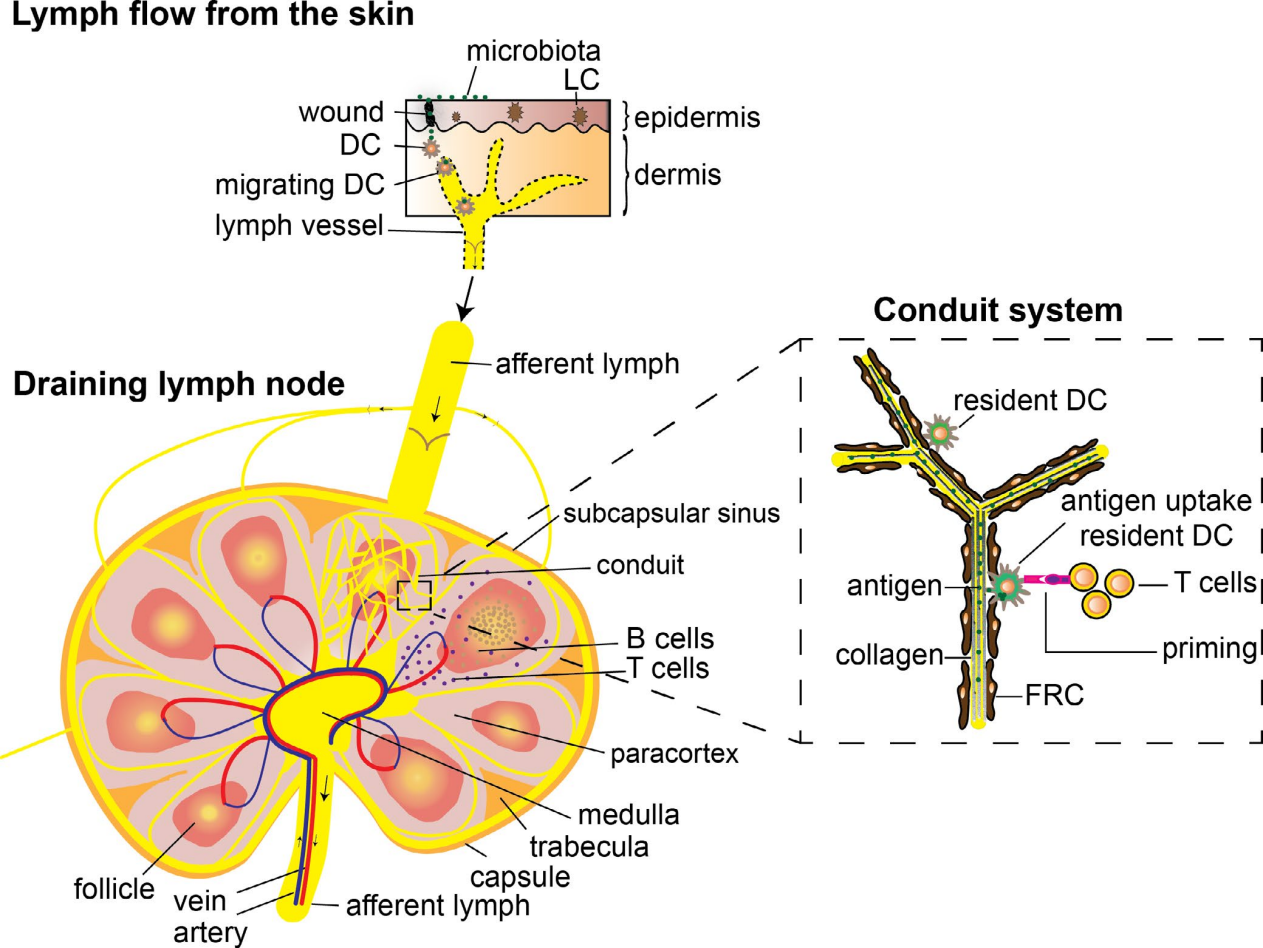
Schematic view of T‐cell activation in a draining lymph node after cutaneous infection. Migrating DC take up microbial antigens at the wound site and route via a lymphatic vessel to a local draining lymph node. A draining lymph node consists of micro‐domains containing paracortical T‐cell areas and follicular B‐cell areas. Lymph fluid (less than 70 kDa) enters the conduit system, which forms a tube system within T‐cell and B‐cell areas. The conduit system consists of organized collagen fibres with FRC wrapped around it. Resident DC within T‐cell areas are able to pick up antigens that flow through the conduit system. Resident mature DC present antigen to local naïve T cells, resulting in T‐cell priming and activation

Upon cutaneous infection, neutrophils produce chemokines that attract immature DC to help antigen clearance. DC are mobile sentinels that operate at the interface between innate and adaptive immunity. In human skin, DC are sub‐classified into Langerhans cells, which reside exclusively in the epidermis, and interstitial dermal DC, which reside in the adjacent dermis.^30^ When immature DC take up pathogens at the wound site, they process them, maturate and migrate to the local draining lymph node (Figure 1).^35^ This migration is mediated by up‐regulation of CCR7 chemokine receptor expression in maturating DC and by the lymphatic vessel and the LN‐derived secondary lymphoid chemokine, CCL21.^36^ High levels of CCL21 secreted by high endothelial venules also recruit CCR7^+^‐naïve and central memory T cells to the T‐cell zones of the draining lymph node.^37^ Generally, migratory DC that have taken up pathogens reach the draining lymph node within 24 h and peak around 2–4 days after infection.^38^ Within the draining lymph node, resident DC maturate and present processed pathogen‐derived antigens onto major histocompatibility complex (MHC) molecules; express co‐stimulatory molecules (CD80 and CD86); and produce inflammatory cytokines. Subsequent interaction with naïve T cells results in the induction of VDJ recombination, development of MHC‐antigen‐specific T‐cell receptors, and priming and activation of T cells or differentiation to central memory T cells.^30^, ^39^

Two major types of T‐cell‐mediated immune responses are initiated by professional antigen‐presenting cells like DC, depending on antigen processing and presentation via MHC class I or MHC class II mechanisms. MHC class I antigen presentation activates cytotoxic CD8^+^ T cells, whereas MHC class II antigen presentation activates CD4^+^ T helper cells. Peptides loaded onto MHC class I molecules are processed cytosolic intracellular antigens. Peptides processed for loading onto MHC class II molecules are processed extracellular antigens that have entered the lysosomal degradation pathway.^40^

Primed CD4^+^ T cells can also differentiate into T cells that secrete IL‐17 (Th17), which is crucial for neutrophil recruitment and antimicrobial response. In line with this, EBS‐derived blister fluid has been shown to contain high levels of Th17 cytokines, which may explain the dense epidermal neutrophilic infiltrate.^22^ Th17 signalling is also persistently activated in immune‐mediated inflammatory diseases, such as psoriasis,^41^ rheumatoid arthritis and inflammatory bowel disease.^42^ It would be interesting to know whether Th17 polarization is also upregulated in RDEB, on the assumption that therapeutic treatment of RDEB patients with anti‐IL‐17 antibodies may have potential.

Besides DC, neutrophils and macrophages can also play a role in adaptive immunity. Upon cutaneous wound infection, neutrophils can travel from the infected site to the nearest draining lymph node, where they can undergo apoptosis. Subsequently, resident DC can present neutrophil‐derived antigens to lymph node resident naive T cells.^43^ Furthermore, macrophages and neutrophils can directly transfer antigens to DC^30^ and present extracellular antigens in the MHC class II‐restricted mechanism. Previously, we have shown that established and chronic RDEB wounds contain equal numbers of Langerhans cells and DC.^7^ Furthermore, we have found that 50% of RDEB chronic wound‐derived DC express CD80^+^CD86^+^ activation markers,^7^ indicating that these wound‐derived mature DC could acquire bacterial wound‐associated antigens and activate T‐cell‐mediated immune response.

## 3 THE ROLE OF T‐CELL‐MEDIATED IMMUNITY IN EB

### 3.1 Central T‐cell education

T‐cell progenitors originate from bone marrow progenitors that migrate to the thymus for selection and education. This process depends on a diverse array of interactions with functionally distinct epithelial cell types within the thymic stroma. Unlike epithelial sheets arranged on a basement membrane of the skin, thymic epithelial cells are organized into a three‐dimensional network. This mesh‐like arrangement of epithelial cells facilitates T‐cell migration and the interaction necessary for T‐cell education.^15^, ^44^, ^45^, ^46^, ^47^

**FIGURE 2:**
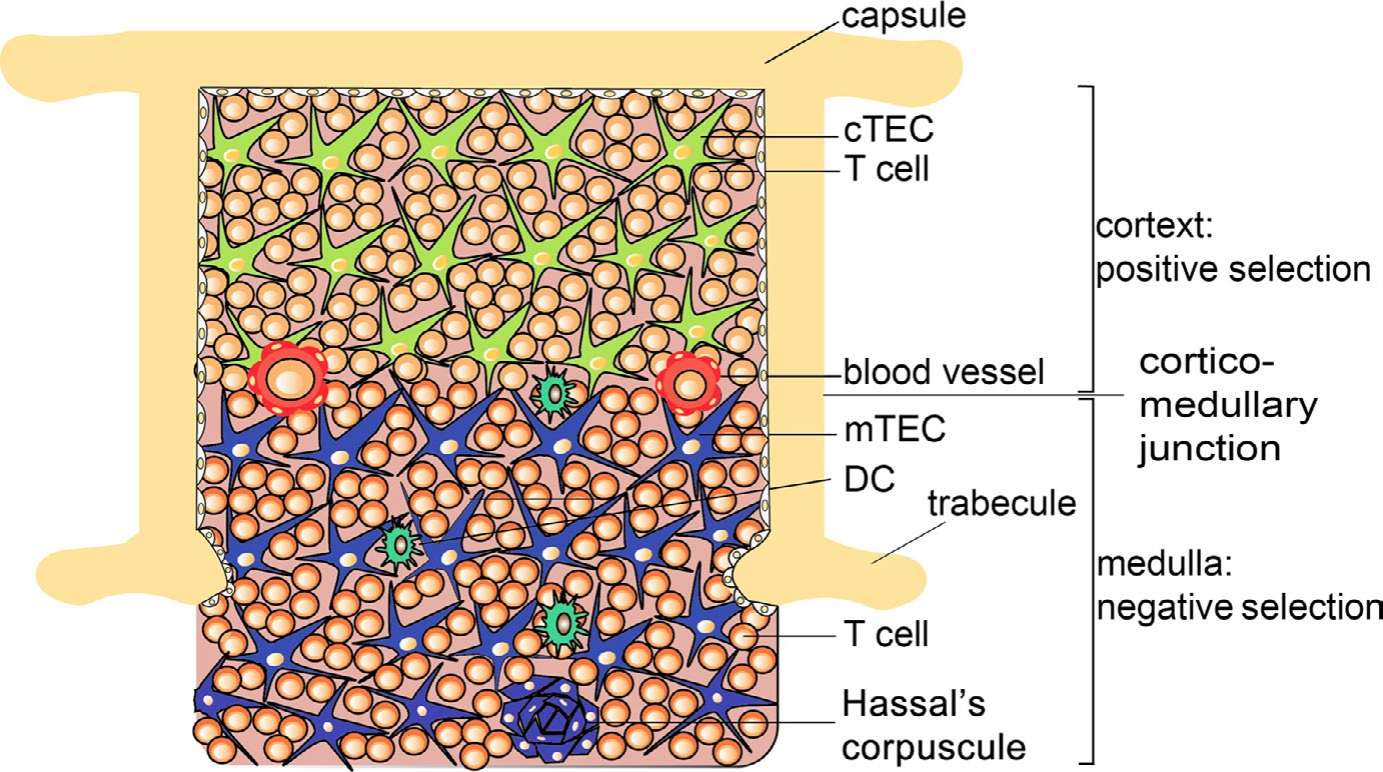
Central T‐cell education in the thymus. The thymic microenvironment directs T‐cell differentiation. T cells that enter the outer cortex undergo positive selection (cTEC, cortical thymic epithelial cells). Next, T cells migrate to the inner medullary area where they undergo negative selection (mTEC, medullary thymic epithelial cells). T cells that survive all educational steps become naïve T cells and migrate to peripheral organs, such as the draining lymph nodes, mucosal barrier sites and exocrine glands

In the thymic outer cortex, double‐positive CD8^+^CD4^+^ T cells are presented to self‐antigens on MHC molecules on cortical thymic epithelial cells. During this positive selection process, T cells that interact with MHC‐I or MHC‐II receive a survival signal. T cells that do not interact die by neglect. Double‐positive T cells that interact with MHC‐I become CD8^+^ T cells, and those that interact with MHC‐II become CD4^+^ T cells.^44^, ^45^ T cells that survive positive selection (around 10%) migrate towards the inner thymic medulla, where they are exposed to a negative selection process. In the medulla of the thymus, T cells are exposed to tissue‐specific self‐antigens on MHC molecules on medullary thymic epithelial cells.^48^, ^49^ Expression of tissue‐specific self‐antigens in medullary thymic epithelial cells is regulated by the transcription regulator AIRE, which activates the expression of proteins found in the periphery, resulting in subsequent loading of tissue‐specific self‐antigens onto MHC molecules.^48^ T cells that bind too strongly to self‐antigens presented by medullary thymic epithelial cells receive an apoptotic signal and die. T cells that bind at intermediate strength levels to self‐antigen deviate into CD4^+^CD25^+^ regulatory T cells (Treg).^44^, ^45^ Expression of the transcription factor forkhead P3 (FOXP3) is a master regulator of Treg development.^50^ Generation of thymus‐derived Treg is important to suppress peripheral abnormal or excessive immune responses.^44^, ^45^ All other T cells that survive the selection exit the thymus as self‐tolerant naïve CD8^+^ or CD4^+^ T cells (Figure 2). When the negative selection process fails, autoreactive T cells are able to enter the periphery, and these autoimmune cells have the potential to react to particular self‐antigens.^48^ Altogether, under physiological conditions, about 98% of T cells die during the entire educational process within the thymus. When educated immunocompetent naïve T cells emigrate from the thymus into the peripheral circulation, they populate organs, such as the draining lymph node, exocrine gland, mucosal barrier sites and even the brain. The majority of naïve T cells are found in lymphoid tissues (such as the spleen, tonsils and estimated 500–700 draining lymph nodes) and large numbers are also found in mucosal sites (such as the intestines and lung) and the skin. Only 2% of the total T‐cell population is found in the peripheral bloodstream.^46^, ^51^

### 3.2 The role of EB‐associated genes in central T‐cell education

**FIGURE 3:**
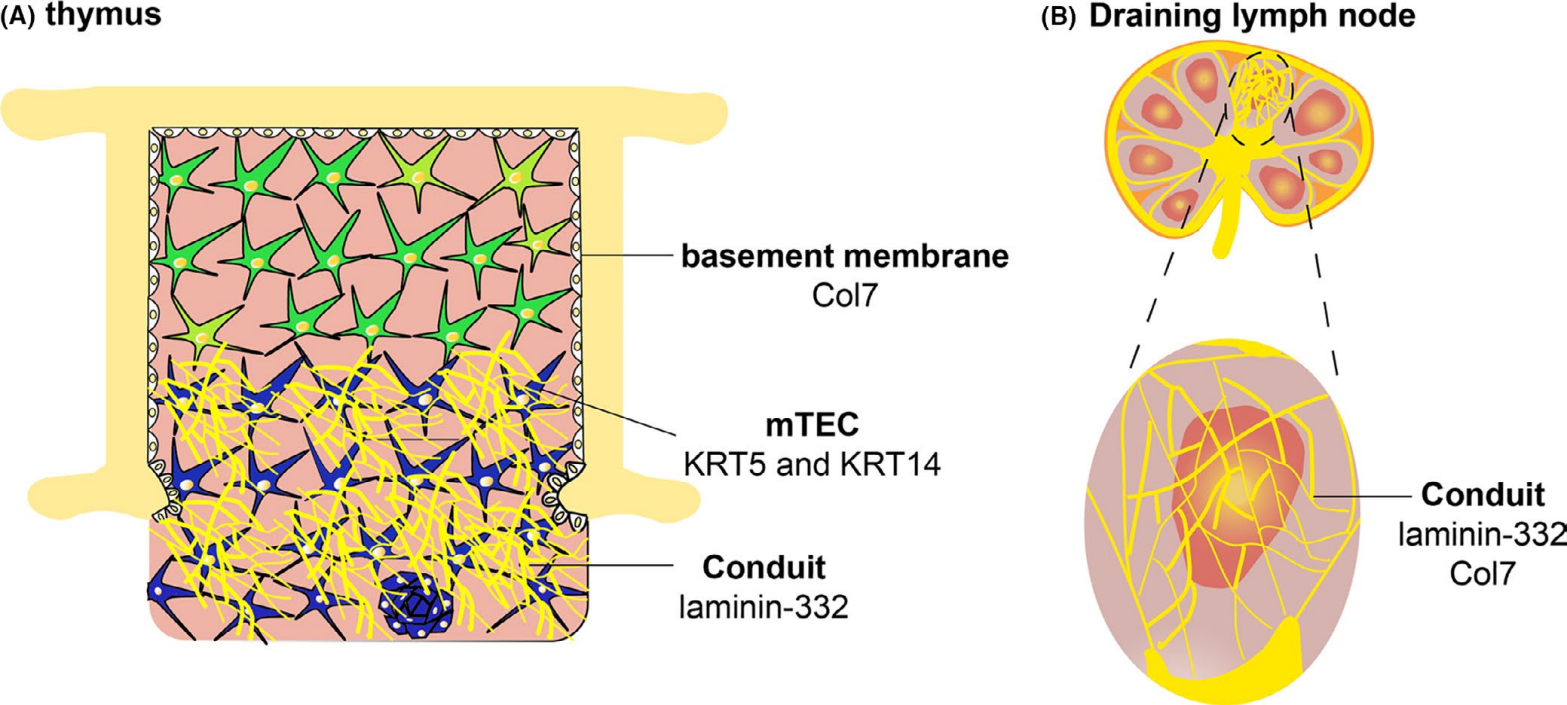
Expression of common EB‐causing gene variants in thymus and draining lymph node (cTEC, cortical thymic epithelial cell and mTEC, medullary thymic epithelial cells). (A) Thymus. (B) Draining lymph node

Epithelial cells synthesize keratins (KRT), which ultimately form intermediate filaments. Functionally, keratin intermediate filaments provide structural support and regulate growth, proliferation, migration and apoptosis.^52^ EBS patients fail to generate proper KRT5 or KRT14 protein, resulting in a skin blistering phenotype.^53^ However, KRT5 and KRT14 are also expressed in medullary thymic epithelial cells in the thymus (Figure 3A).^49^ This suggests that EBS patients may have an immunological defect in central tolerance due to a structural defect in the thymus, affecting T‐cell education. This could subsequently result in defective peripheral T cells that may fail to clear cutaneous wound infections.

The basement membrane protein laminin‐332 is a heterotrimeric molecule comprising α3 (respective gene is *LAMA3*), β3 (respective gene is *LAMB3*) and γ2 (respective gene is *LAMC2*) subunits.^2^ Mutations in either of the genes encoding laminin‐332 are responsible for weak dermal‐epidermal junction and skin fragility in JEB patients.^2^ However, laminin‐332 is also expressed in the medulla of the thymus (Figure 3A).^16^, ^54^, ^55^ In the skin, laminin‐332 is important for the formation of tissue‐stabilizing hemidesmosomes, which bind the epidermis to the underlying dermis.^2^ Therefore, it seems likely that this extracellular matrix protein has a similar structural function in the thymus. Furthermore, it has been shown that CD8^+^ T cells bind laminin‐332 via integrin α6β4, and this interaction promotes T‐cell migration.^54^, ^56^ It has been also demonstrated that soluble laminin‐332 inhibits the proliferation of T cells induced by anti‐CD3 plus IL‐2 in vitro.^55^ Therefore, a functional defect of laminin‐332 in the medulla in the thymus strongly suggests that there is an intrinsic defect in T‐cell‐mediated immunity in JEB patients, which may consequently affect wound healing.

Hereditary RDEB is caused by mutations in the *Col7A1* gene, resulting in lack or dysfunction of Col7.^2^ Col7 is expressed by keratinocytes and dermal fibroblasts at the dermal‐epidermal junction, where it plays an important structural role in the formation of anchoring fibrils.^2^ Imaging studies have shown that Col7 is also expressed in the thymic basement membrane of the capsule (Figure 3A).^57^ This suggests that its dysfunction may affect the three‐dimensional structure of the thymus, which may consequently affect proper T‐cell education.

To date, no data regarding T‐cell education in EB patients are available. Therefore, it would be interesting to investigate the neonatal thymic architecture, T‐cell development and wound healing in EB‐recapitulating transgenic animals to predict whether a lack of KRT5 and/or KRT14 or laminin‐332 or Col7 affects T‐cell‐mediated immunity.

### 3.3 T‐cell activation in peripheral draining lymph nodes

Peripheral targeted naïve T‐cell activation to non‐self‐pathogenic antigens generally occurs in the highly organized peripheral draining lymph node. These lymphoid organs are strategically positioned throughout the body.^58^ The architecture of the draining lymph node is characterized by distinct micro‐domains for T cells, which provide a favourable microenvironment for efficient T‐cell activation. The micro‐domains within the draining lymph node are structured by stromal cells, which secrete important molecules that ensure attraction, retention and survival of T‐cell subsets within the separate regions (Figure 1). Importantly, a disturbed architecture of these micro‐domains in the draining lymph node correlates with reduced immune competence.^58^, ^59^

Stromal cells that associate in the paracortex with T cells in a draining lymph node are fibroblastic reticular cells (FRC). FRC produce reticular fibres that form a dense conduit network within a draining lymph node. Structurally, the conduit system (or lymphoid extracellular matrix) consists of a collagen tubular network that forms a three‐dimensional reticulum (like a sponge), where lymphocytes fill up the compartments. FRC producing the extracellular matrix of the conduit system are wrapped around the highly organized core collagen fibres surrounded by a basement membrane (Figure 1).^59^, ^60^

Free antigens of less than 70 kDa can be drained from afferent lymphatics into the conduit system. In lymphoid T‐cell micro‐domains, these conduits align resident DC that can move and capture antigens within the lumen of conduits so that they can be presented to local T cells (Figure 1). Of note, larger antigens are probably removed from the incoming lymph by a large population of macrophages lining the outer border of the lymphoid compartment (subcapsular sinus) by currently unclear mechanisms.^60^ Within lymphoid compartments, naïve T cells that recognize resident DC‐presented antigens are activated, resulting in local IL‐2 production, proliferation and differentiation to effector T cells. Effector T cells can subsequently migrate via blood vessels to the peripheral tissues to promote pathogen clearance.^46^

After an inflammatory response, a proportion of activated effector T cells differentiates into central memory T cells. The presence of memory T cells in the skin is important to ensure a quick and adequate response to invasive pathogens in case of a barrier breach.^61^, ^62^ Memory T cells can be activated without activating co‐stimulatory molecules or antigen presentation on antigen‐presenting cells. Furthermore, memory T cells have the potential to maintain long‐term immunity towards a specific non‐self‐antigen derived from a particular pathogen.^46^

Recently, we have demonstrated that up to 80% of all T cells in early RDEB wounds are represented by activated memory T cells,^7^ suggesting that former pathogenic microorganisms are trying to invade again when the skin barrier is breached. However, we have also observed that CD45RO^+^ effector memory T cells differentiate into CD45RA^+^ effector T cells when wounds are transitioning from early to chronic state.^7^ Changes in T‐cell populations during RDEB wound progression are currently under investigation.

Next to the paracortex containing T cells, the conduit system is also present within the B‐cell follicle domain in draining lymph nodes. These areas are also rich in follicular DC (FDC).^63^ It has been shown that Col7 is primarily present in the conduit system in the follicular B‐cell zone and in close association with FDC in the spleen, but not in draining lymph nodes (where Col7 is also expressed outside the B‐cell areas, Figure 3B).^12^ In the lumen of the conduit system, Col7 binds the innate immune activator cochlin. As described in the “macrophages section” above, the cochlin‐col7 axis in lymphoid organs is important for effective innate immunity against pathogenic microbiota. Therefore, mechanistic functions of the conduit system in lymphoid organs, both in general as well as in RDEB, require further investigation.

## 4 MICROBIOTA INTERFERING IN EB WOUND HEALING

The human skin acts as a physical barrier and the first line of defense against the invasion of pathogenic microorganisms.^64^ Structurally, the outer layer of the epidermis consists of terminally differentiated, enucleated (squamous) keratinocytes that are chemically cross‐linked to provide a barrier.^65^, ^66^ This cutaneous barrier restricts water loss and the entry of potentially harmful substances.^35^, ^64^, ^67^ From an immunological perspective, the skin qualifies as an innate immune surveillance system.^68^, ^69^ The outer epidermal surface of the skin, although serving as an antimicrobial shield, is home to millions of different environmental microorganisms.^69^

Humans (like all vertebrates) enter the world germ‐free, and microbial communities colonize the skin immediately after birth. Postnatally, diverse microbial communities colonize the skin and shape the immature immune system, resulting in the development of mature immune‐commensal homeostasis.^70^ Consequently, most environmental microbiota are non‐pathogenic for immunocompetent hosts. Importantly, cutaneous microbiota become beneficial and supply vitamins for the skin, and/or prevent the colonization of pathogens by producing antimicrobial peptides to maintain healthy skin. However, when the host's immune system develops incompletely after birth (such as from a potential defect in T‐cell education in EB patients), immune‐commensal homeostasis may fail to develop. Consequently, commensal microbes can become pathogenic and result in a shift in microbial communities on the diseased host (termed dysbiosis).^65^ Dysbiosis has been shown in other skin diseases, such as atopic dermatitis, psoriasis and acne.^71^

When the skin barrier is breached due to a scratch or wound, skin commensal microorganisms gain access to richer, blood‐derived nutrients and can colonize and invade an open wound.^30^ Within a wound, commensal bacteria can become opportunistic and increase in numbers. This results in a rapid induction of the innate immune response of the host to prevent further pathogenic proliferation and expansion.^65^ Furthermore, the presence of memory T cells, particularly CD4^+^ T helper cells in the skin, supports innate immunity by secreting IFNγ to facilitate a quick response towards a recurrent pathogenic microorganism.^51^ When pathogenic microorganisms prevail over innate immunity, adaptive immunity can further combat infection.^30^ It is important that an adaptive immune response towards wound‐colonizing opportunistic bacteria is carefully orchestrated so that pathogenic microorganisms can be destroyed while avoiding an excessive immune response that may harm the host.^68^

Repetitive, mechanically induced cutaneous lesions predispose EB patients to bacterial infection.^9^ Areas of denuded skin increase the risk for cutaneous infections due to the exposure of body fluids to the microbial environment.^72^ The interaction between cutaneous wounds and microbiota can generally be divided into 4 stages: contamination, colonization, critical colonization and infection.^9^, ^73^ Generally, all chronic wounds are considered to be contaminated with environmental non‐replicating microorganisms.^74^ Under healthy circumstances, these contaminants do not interfere with wound healing, and they are quickly cleared by the host's innate immunity. A wound is considered colonized when opportunistic microorganisms adhere to the wound bed and start replicating. At this stage, wound healing is still not affected.^75^ However, during the critical colonization stage, opportunistic microorganisms become pathogenic and invasive, interfere with wound healing and trigger the inflammatory response.^1^, ^75^ This can be recognized clinically by the presence of erythema, warmth, swelling, odour and pain at the local wound area.^1^, ^7^ If the host immune system fails to fight the cutaneous infections, this stage can progress to systemic infection and ultimately sepsis. Importantly, sepsis is a leading cause of infant mortality in EB patients.^8^, ^9^ Besides sepsis, these chronically infected wounds can give rise to a highly aggressive form of SCC, which develops in more than >90% of RDEB patients by the age of 55 years.^10^, ^11^

Common wound colonizers in EB patients are mainly opportunistic bacteria, such as *Staphylococcus aureus*, *Streptococci* and *Pseudomonas aeruginosa*.^9^, ^76^ *Staphylococcus aureus* is the predominant species isolated from cutaneous EB‐derived wound culture samples.^1^, ^30^ There is a great deal of genetic variation of *Staphylococcus aureus* species, and wounds from EB patients can be colonized with multiple types of *Staphylococcus aureus*. A distribution map of the most prevalent molecular *Staphylococcus aureus type* in different continents has been generated.^77^ Furthermore, molecular typing of *Staphylococcus aureus* isolates derived from EB patients and their healthcare workers located in the Netherlands show that the population structure of EB‐derived *Staphylococcus aureus* mirrors the general *Staphylococcus aureus* population structure of this country.^1^, ^30^, ^78^

The most important factor that determines whether opportunistic microorganisms infect cutaneous wounds is the state of the host immune response.^67^, ^75^ A successful immune response can be affected by many variables, such as diabetes, alcohol use, malnutrition, ageing and an underlying systemic immune disorder.^30^ Since most common EB‐related gene variants are also expressed in lymphoid organs,^12^, ^15^, ^16^ a high predisposition of cutaneous infections in EB patients may be due to their T‐cell‐mediated immune defect that consequently affects antibacterial immunity. Importantly, it has been shown in a mouse model with conditional deletion of collagen 7 that increased bacterial colonization precedes reduced skin integrity and wounds.^12^ This strongly indicates that the increased susceptibility to bacteria in EB patients is primarily due to an antibacterial immunity defect. Therefore, mechanistic functions of EB‐related gene variants in T‐cell‐mediated immunity in lymphoid organs need to be further investigated. Once more fundamental scientific knowledge is learned, effective interference for clinical therapy can be considered.

## 5 FUTURE PERSPECTIVES

There is no cure for EB, and painful chronic wounds are one of the major complications.^17^ EB is treated with topical antiseptics or topical antibiotics. When this treatment fails and wound infection is clinically diagnosed, EB patients are treated with systemic antibiotics based on bacterial culture results.^72^ However, bacterial resistance, such as *Staphylococcus aureus* resistance to antibiotics, is rising, posing a serious concern.^30^

As discussed in this review, there is evidence that RDEB patients have an underlying immunity defect that affects antibacterial immunity. Therefore, therapeutic targeting of systemic inflammatory mediators, in combination with regenerative medicine and/or conventional antibiotics, could be an effective therapeutic strategy to alleviate disease manifestations. Based on the current immunological knowledge of RDEB, neutrophil recruitment towards wound areas should be regulated; this recruitment can be achieved by CXCR2 antagonists.^25^, ^28^ Alternatively, anti‐IL‐17 antibodies may hold potential utility since Th17 cytokines important for neutrophil recruitment are upregulated in EBS blister fluid.^22^ Because neutrophils are a very important first‐line defense against pathogenic microorganisms, regulating their recruitment may accelerate wound healing in patients. Furthermore, additional knowledge is required about the phagocytic activity of neutrophils present in RDEB patients compared with healthy individuals.

A promising novel therapeutic strategy is systemic administration of the cochlin LCCL domain, which has been shown to decrease cutaneous bacterial colonization in RDEB mice.^12^ Also, the cochlin LCCL domain has been shown to activate macrophages,^12^ and a low percentage of macrophages has been found in chronic RDEB wounds.^7^ Therefore, additional studies are required about the activity of RDEB‐derived macrophages compared with healthy individuals. Additional scientific knowledge is also needed about the immune‐modulating effect of the cochlin LCCL domain on RDEB‐derived macrophages.

From a fundamental immunological perspective, the functional role of EB‐related gene variants in lymphoid tissues should be studied in further detail, as well as the functional role of the conduit system. This knowledge will not only contribute to new insights about the EB phenotype, but it is also important to further understand a broad range of diseases wherein lymphoid tissues are involved. As EB‐related gene variants are also expressed in the thymus, further understanding is needed about the role of EB‐derived T cells in maintaining chronic wounds as well as about the preservation of antibacterial immunity defects.

Taken together, the evidence presented in this review provides strong evidence that there is systemic inflammatory involvement hidden below mucocutaneous manifestations in RDEB patients. The ability to regulate chronic inflammation and antibacterial immunity of cutaneous wounds in EB patients would improve their quality of life as well as their life expectancy.

## CONFLICT OF INTEREST

The authors declare no conflict of interest.

## AUTHOR CONTRIBUTIONS

LH, TP, VA and OI participated in writing, discussion and revising the paper, and have all read and approved the final manuscript.

## Data Availability

The data that support the findings of this study are openly available.
